# Recent advances in photodynamic therapy systems based on pillar[n]arene host–guest chemistry

**DOI:** 10.3389/fchem.2026.1924748

**Published:** 2026-08-12

**Authors:** Fuqiang Zhao, Zhiying Zhu, Liang Li

**Affiliations:** School of Chemical and Environmental Engineering, Shanghai Institute of Technology, Shanghai, China

**Keywords:** host-guest chemistry, photodynamic therapy, photosensitizers, pillar[n]arene, reactive oxygen species

## Abstract

Photodynamic therapy (PDT) is a minimally invasive and low-toxicity strategy for tumors and bacterial infections. However, conventional small-molecule photosensitizers have poor water solubility, severe aggregation-caused quenching and weak tumor targeting, restricting their clinical translation. Macrocyclic host-guest supramolecular assembly improves the performance of photosensitizers via reversible noncovalent recognition. Pillar[n]arenes feature symmetric electron-rich cavities, abundant modifiable sites and wide guest compatibility, showing outstanding advantages over cyclodextrins, calixarenes and cucurbiturils for responsive photosensitizing platforms. This review summarizes recent advances of pillar[n]arene host-guest systems in PDT. We introduce the structural and recognition features of pillar[n]arenes, classify the fabrication of pillar[n]arene-photosensitizer complexes, and interpret how host-guest inclusion alleviates fluorescence quenching, elevates singlet oxygen yield and triggers tumor microenvironment-responsive drug release. Their applications in anti-tumor PDT, antibacterial photodynamic disinfection and imaging-guided therapy are outlined. We also discuss translational obstacles including insufficient biocompatibility and limited deep-tissue penetration, and propose future directions involving targeted functionalization, multi-modal synergy and degradable macrocyclic skeletons. This work clarifies the structure-activity relationship of pillar[n]arene-based PDT systems and offers guidelines for designing high-performance supramolecular theranostic agents.

## 1 Introduction

Photodynamic therapy (PDT) is a minimally invasive optical treatment modality based on photosensitizers (PSs), offering spatiotemporal controllability and precision therapeutic capability (Chen et al., 2020; Shen et al., 2022; Xie et al., 2024; Zhou et al., 2021). In the ground state, PSs are nontoxic; upon irradiation with a specific wavelength, they undergo excitation to a singlet state, followed by intersystem crossing to a longer-lived triplet excited state. Subsequently, energy and electron transfer between the excited PSs and ambient oxygen or endogenous biomolecules generate reactive oxygen species (ROSs), such as singlet oxygen (^1^O_2_), hydroxyl radicals (•OH), and superoxide anions (O_2_^−^), which induce oxidative stress-mediated destruction of pathological cells while sparing adjacent healthy tissues (Gong, et al., 2025; Qi et al., 2024; Zou et al., 2024a; Zou et al., 2024b). Clinically, PDT is typically administered in two steps: first, systemic or topical delivery enables preferential accumulation of PSs at target lesions; second, localized light exposure activates the enriched PSs to produce ROS, achieving efficient ablation of diseased tissues (Cai R. et al., 2025; Tao et al., 2025; Wang, et al., 2025c; Zou et al., 2025). To date, PDT has been broadly applied in the management of malignancies, drug-resistant infections, and degenerative ocular disorders, positioning it as a promising therapeutic alternative to conventional surgery, radiotherapy, and chemotherapy (Chen et al., 2026; Jin et al., 2021; Li L. et al., 2025; Liu X. et al., 2025; Wu et al., 2024). Driven by the growing emphasis on precision and minimally invasive medicine, the development of intelligent PDT platforms with enhanced safety, responsiveness, and targeting performance has emerged as a focal research direction at the interface of optical engineering, functional materials, and biomedical engineering (Cai et al., 2021; Wang et al., 2022a; Wang et al., 2022e; Wang et al., 2023b; Zhang et al., 2021; Zhou et al., 2023).

Compared with conventional surgery, chemotherapy, and radiotherapy, PDT offers four distinctive advantages: minimal invasiveness, exceptionally low systemic toxicity, absence of therapeutic resistance, and high versatility for combination with other treatment modalities (Dai et al., 2025; Deng, et al., 2026; Liu T. et al., 2025; Tang et al., 2024; Wu et al., 2025). Despite these merits, traditional small-molecule PSs suffer from inherent limitations, including poor water solubility, insufficient tumor accumulation, nonspecific phototoxicity to normal tissues, and aggregation-caused quenching (ACQ), which substantially hinder their clinical translation (Lan et al., 2019; Kessel, 2019; Pham et al., 2021; Zhang T. et al., 2025; Cai et al., 2025b). To address these challenges, various modification strategies have been developed, such as conjugation of hydrophilic moieties, extension of π-conjugation, introduction of electron-donating groups for red-shifted absorption, coupling with targeting ligands, and encapsulation in nanocarriers (Chen X. et al., 2024; Wang J. et al., 2023; Zhao et al., 2023). Among these, supramolecular self-assembled nano-PSs constructed via noncovalent interactions have emerged as a particularly attractive platform. Such systems leverage the enhanced permeability and retention (EPR) effect for passive tumor targeting, enable stimuli-responsive disassembly in the tumor microenvironment, afford red-shifted absorption for enhanced deep-tissue penetration, and allow modular co-assembly with diverse therapeutic moieties to construct integrated theranostic platforms (Lin et al., 2022; Wang X. et al., 2022; Xu et al., 2022). Although several reviews have summarized advances in self-assembled supramolecular PSs, a dedicated overview focusing on macrocycle-mediated precision recognition for PDT remains lacking (Kwon et al., 2021; Li S. et al., 2025; Yang et al., 2020).

Macrocyclic host–guest chemistry represents a core discipline within supramolecular chemistry, relying on specific molecular recognition between macrocyclic hosts and size- and geometry-matched guest molecules to enable reversible assembly and functional regulation (Cao et al., 2021; Chen F.-Y. et al., 2024; Tang et al., 2023; Wang F. et al., 2025). The five most widely applied families of classical macrocyclic hosts—crown ethers, cyclodextrins, calixarenes, cucurbiturils, and pillar[n]arenes—possess intrinsic cavity structures that facilitate the precise encapsulation of complementary guests through synergistic noncovalent interactions, including hydrophobic effects, hydrogen bonding, electrostatic forces, and van der Waals contacts (Dong et al., 2023; Ezquerra Riega et al., 2024; Saddik et al., 2020; Shukla et al., 2023). By virtue of their size complementarity and polarity adaptability, macrocyclic host–guest complexes exhibit unique advantages such as precise controllability, dynamic reversibility, and sensitive environmental responsiveness (Chen et al., 2023; Feng et al., 2020; Han et al., 2020; Wu et al., 2021; Zhang et al., 2024). In recent years, supramolecular theranostic platforms constructed on the basis of host–guest recognition have achieved remarkable progress in precision diagnosis and targeted therapy of diseases. Compared with traditional covalent modification strategies, macrocycle-mediated assembly offers simpler preparation protocols, enhanced structural tailorability, and intrinsic dynamic reversibility, rendering it particularly amenable to the complex and dynamic *in vivo* physiological micro-environment (Li H. et al., 2025; Li Q. et al., 2025; Wang et al., 2023c; Wang R.-P. et al., 2024; Zhou et al., 2024). Consequently, macrocycle-based supramolecular PSs have garnered considerable research interest in the field of intelligent PDT, where spatiotemporal control over PS activation is critically demanded. The modular and noncovalent nature of this approach further facilitates the integration of multiple therapeutic and imaging functions, thereby addressing the inherent heterogeneity of diseases and offering a versatile platform for advancing precision medicine (Chen et al., 2022; Ding et al., 2023; Wang et al., 2022d; Yu et al., 2024).

**SCHEME 1:**
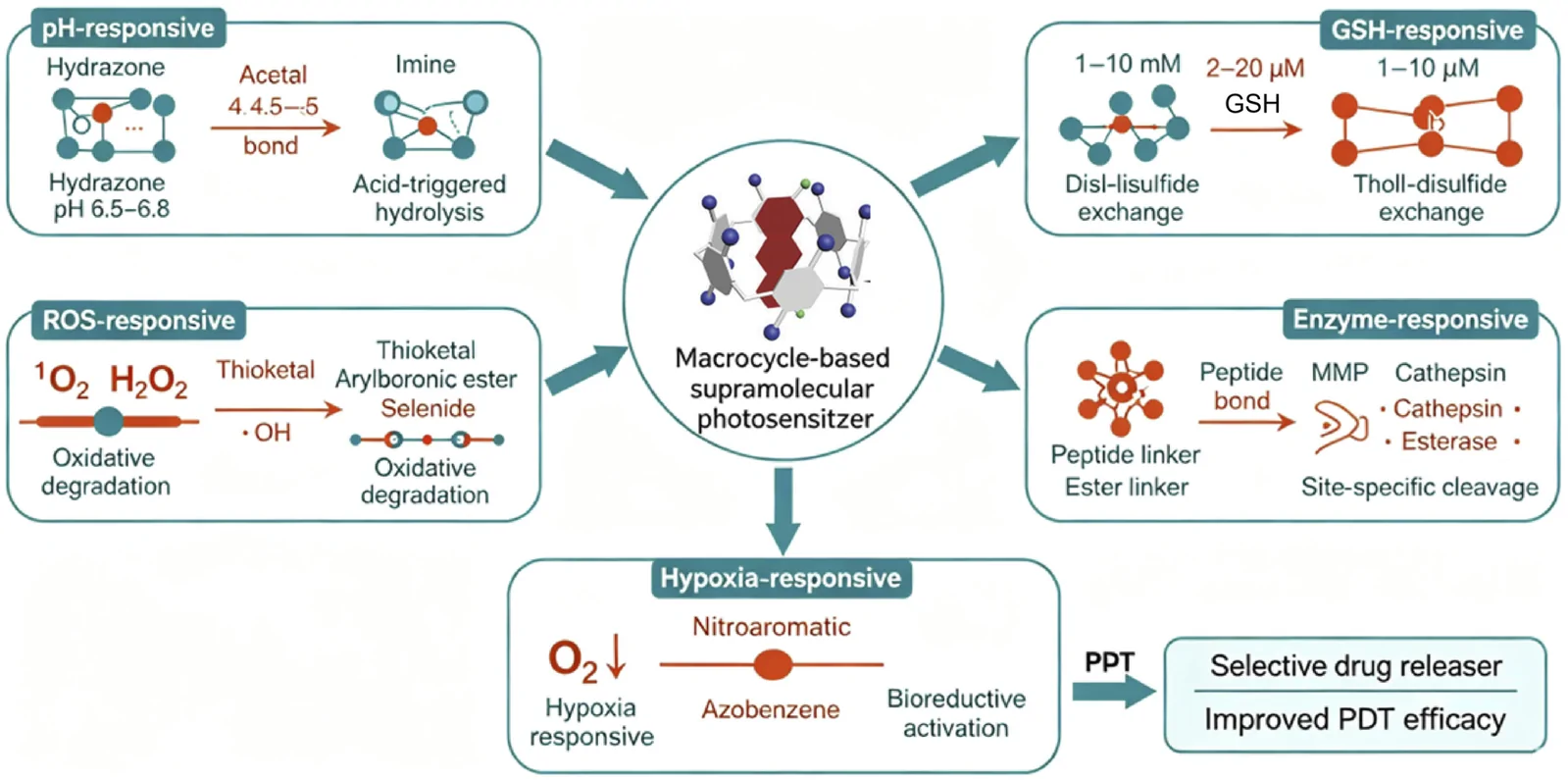
Common microenvironment-adaptation logic of macrocycle-mediated supramolecular photosensitizers.

To rationally design macrocycle-based supramolecular photosensitizers for intelligent PDT, it is essential to recognize that different stimuli-responsive systems are unified by a common microenvironment-adaptation logic. As illustrated in Scheme 1, each triggering mechanism corresponds to a specific pathological feature of the tumor microenvironment: (1) pH-responsive systems exploit the endosomal/lysosomal acidity (pH 4.5–6.5) and the mildly acidic tumor extracellular milieu (pH 6.5–6.8), typically employing acid-labile linkers such as hydrazones, acetals, or imines that undergo hydrolysis under low-pH conditions; (2) glutathione (GSH)-responsive systems target the high intracellular GSH concentration (1–10 mM in cytosol versus 2–20 μM in extracellular fluids), commonly utilizing disulfide bonds that are cleaved by thiol–disulfide exchange reactions, enabling selective drug release in the reducing intracellular environment; (3) ROS-responsive systems are designed for inflammatory or PDT contexts where elevated reactive oxygen species (e.g., ^1^O_2_, O_2_^−^, •OH) are present, employing ROS-cleavable linkers such as thioketals, arylboronic esters, or selenides that undergo oxidative degradation; (4) enzyme-responsive systems leverage the overexpression of specific enzymes (e.g., matrix metalloproteinases, cathepsins, or esterases) in tumor tissues, utilizing enzyme-labile peptide or ester linkages for site-specific drug liberation; and (5) hypoxia-responsive systems capitalize on the low oxygen tension characteristic of solid tumors, employing bioreductive groups such as nitroaromatics or azobenzenes that are reduced under hypoxic conditions. This unified framework reveals a core design principle: the choice of stimuli-responsive motif is dictated by the biochemical signature of the target disease site, rather than by random selection of available chemical handles. Consequently, macrocycle-based supramolecular systems are uniquely positioned to integrate multiple stimuli-responsive modules orthogonally, enabling sophisticated “AND-gate” logic for combinatorial therapy—an embodiment of the microenvironment-adaptation philosophy that represents a paradigm shift from conventional “always-on” therapeutic agents toward intelligent, context-aware nanomedicines.

Pillar[n]arenes, first reported in 2008 as a novel class of macrocyclic hosts (Ogoshi et al., 2008), possess unique structural and host–guest recognition advantages over conventional macrocycles, rendering them ideally suited for constructing intelligent PS delivery systems (Shao et al., 2020; Teng et al., 2022). Unlike calixarenes (cone-shaped asymmetric cavities) or cyclodextrins (truncated cone cavities), pillar[n]arenes feature highly symmetric rigid cylindrical cavities with electron-rich interiors, enabling efficient binding of cationic, electron-deficient neutral, and aromatic guests with superior versatility (Dhara et al., 2026; Ohtani et al., 2025; Sun et al., 2025; Tuo et al., 2026; Wang et al., 2022b). Moreover, the readily modifiable hydroxyl groups at both rims allow convenient incorporation of hydrophilic segments, targeting ligands, responsive moieties, and fluorophores, enabling fine-tuning of cavity polarity, solubility, biocompatibility, and microenvironment responsiveness—a distinct advantage over rigid and poorly functionalizable cucurbiturils, size-limited cyclodextrins, and calixarenes with asymmetric cavities (Behera et al., 2025; Sun et al., 2026; Wang J. et al., 2025; Yan et al., 2026).

These exceptional recognition properties make pillar[n]arenes promising platforms for constructing supramolecular photosensitizers that address key clinical limitations: (i) single-molecule encapsulation suppresses π–π stacking and aggregation-induced quenching, enhancing reactive oxygen species production; (ii) noncovalent assemblies exhibit stimuli-responsive dissociation under tumor microenvironment cues (pH, glutathione, enzymes, ROS), enabling stable circulation and on-demand release at lesions while minimizing off-target phototoxicity; (iii) modular assembly avoids multistep synthesis, integrating photosensitizers, chemotherapeutics, photothermal agents, and targeting units via simple physical mixing for synergistic combination therapy; (iv) morphology and size control via host–guest recognition optimizes pharmacokinetics and tumor accumulation (Wu et al., 2018). To date, pillar[n]arene-based photodynamic systems have been successfully applied to solid tumor ablation, antibacterial photodynamic therapy against drug-resistant infections, imaging-guided precision therapy, and tumor micro-environment-responsive smart drug delivery, demonstrating substantial potential in bio-medicine.

**SCHEME 2:**
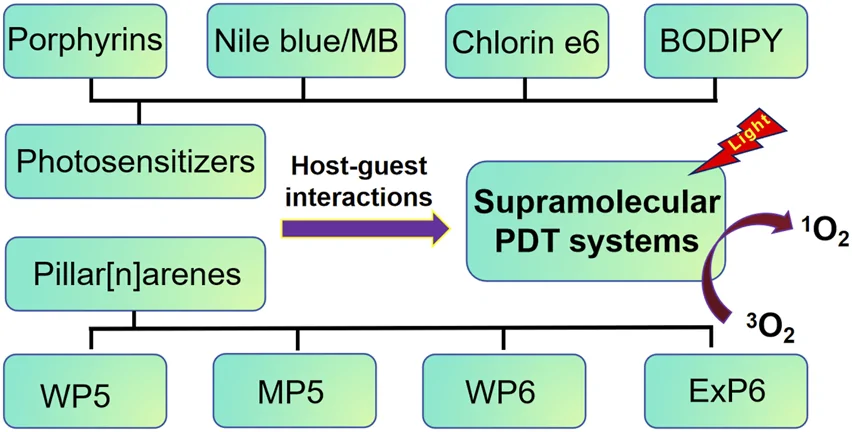
Illustration of supramolecular PDT systems mediated by host-guest complexation between pillar[n]arenes and photosensitizers as discussed in this review.

Although existing reviews have addressed the synthesis, host-guest recognition mechanisms, and broad biomedical applications of pillar[n]arenes (Liu et al., 2026a; Liu et al., 2026b), and several others have overviewed the use of diverse macrocycles in PDT (Sangiamkittikul et al., 2025; Wen et al., 2024), a systematic review dedicated exclusively to the regulation of PDT systems by pillar[n]arene-based host-guest chemistry-elucidating the underlying mechanisms, structural design strategies, and therapeutic scenarios-remains absent. To fill this gap, this Review comprehensively summarizes research advances over the past decade in this area. We categorize the reported pillar[n]arene-based supramolecular photosensitizer systems according to the types of functionalized pillar[n]arene hosts, including water-soluble pillar[5]arenes (WP5), monofunctionalized pillar[5]arenes (MP5), water-soluble pillar[6]arenes (WP6), and extended pillar[6]arenes (ExP6) (Scheme 2). We then dissect the mechanism by which host-guest interactions suppress PS quenching and enable microenvironment-responsive drug release. The discussion further consolidates the application outcomes in two major domains: antitumor PDT and antimicrobial photodynamic inactivation. Finally, we critically assess the key challenges hindering clinical translation, including biosafety concerns, poor *in vivo* accumulation, and limited light penetration depth. Perspectives on the future development of intelligent, precision-targeted, and clinically translatable pillar[n]arene-based supramolecular PDT theranostic platforms are also provided, with the aim of offering comprehensive guidance for the design and development of next-generation macrocyclic supramolecular photosensitive materials. A full name abbreviation cross-reference was shown in Table 1.

**TABLE 1:** Full name abbreviation cross-reference table.

Full name	Abbreviation
Photodynamic therapy	PDT
Photosensitizers	PSs
Reactive oxygen species	ROSs
Aggregation-caused quenching	ACQ
Enhanced permeability and retention	EPR
Glutathione	GSH
Singlet oxygen	^1^O_2_
Hydroxyl radicals	•OH
Superoxide anions	O_2_^−^
Photothermal therapy	PTT
Photoacoustic imaging	PAI
Near-infrared	NIR
Gold nanorods	AuNRs
Scanning electron microscopy	SEM
Transmission electron microscopy	TEM
Lactate oxidase	LOx
Chemodynamic therapy	CDT
Methylene blue	MB
Camptothecin	CPT
Quantum dot	QD
Confocal laser scanning microscopy	CLSM
Supramolecular nanoprodrug	SNP

## 2 Supramolecular PDT systems based on water-soluble Pillar[5]arene host-guest chemistry

**FIGURE 1:**
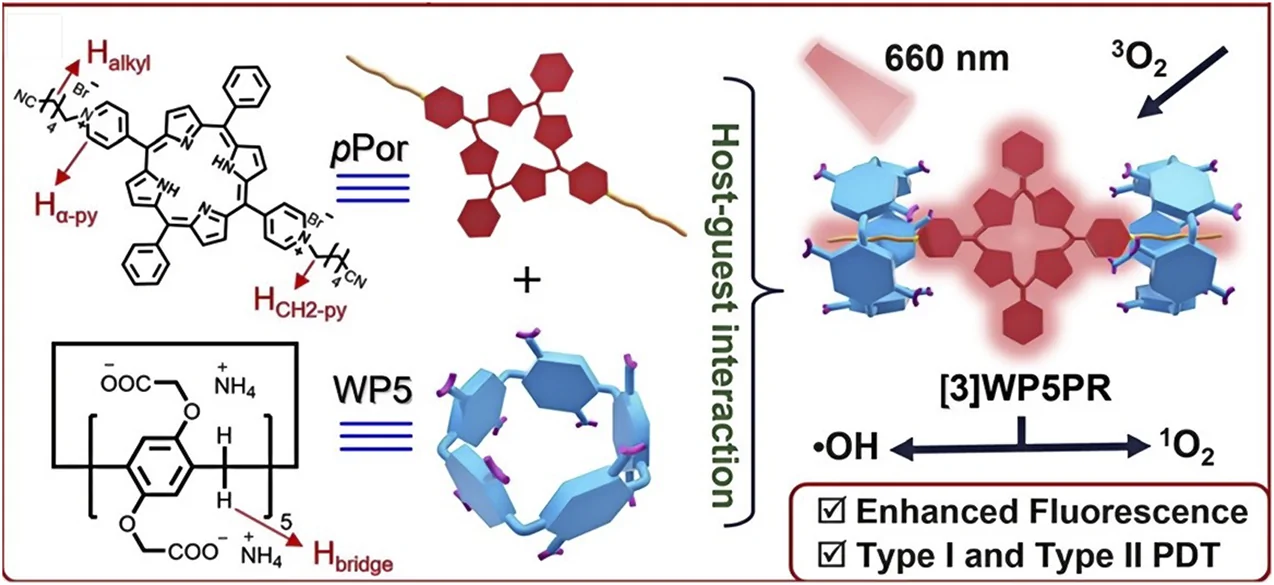
Chemical structures of para-modified porphyrin (pPor) and carboxylatopillar [5]arene (WP5) and schematic illustration of the construction and application of water-soluble [3]pseudorotaxane ([3]WP5PR). Adapted with permission from Zhang et al., 2023.

When water-soluble groups such as carboxylates or quaternary ammonium salts are introduced onto the upper and lower rims of the pillar[n]arene skeleton, the resulting macrocycles exhibit good water solubility (Wang J. et al., 2024). The encapsulation of PSs or guest-modified PSs within the hydrophobic cavity of pillar[n]arenes can effectively improve the solubility of the PSs and inhibit their aggregation, thereby enhancing the efficiency of ROSs generation. In 2023, we successfully constructed a water-soluble [3]pseudorotaxane, designated [3]WP5PR, which exhibits enhanced fluorescence emission (Figure 1). This system was fabricated through host–guest interactions between a para-substituted porphyrin photosensitizer (pPor) and carboxylatopillar[5]arene (WP5), and was further applied to cell imaging and combined type I and type II PDT (Zhang et al., 2023). The introduction of WP5 not only enhances the fluorescence emission of pPor but also induces the formation of stable nanoparticles via amphiphilic self-assembly, facilitating cellular uptake. Importantly, upon irradiation with 660 nm light, [3]WP5PR mediated effective photodynamic cancer therapy by generating OH and ^1^O_2_ through both type I and type II mechanisms. Undoubtedly, [3]WP5PR expands the diagnostic and imaging applications of pseudorotaxanes in cells and microorganisms.

**FIGURE 2:**
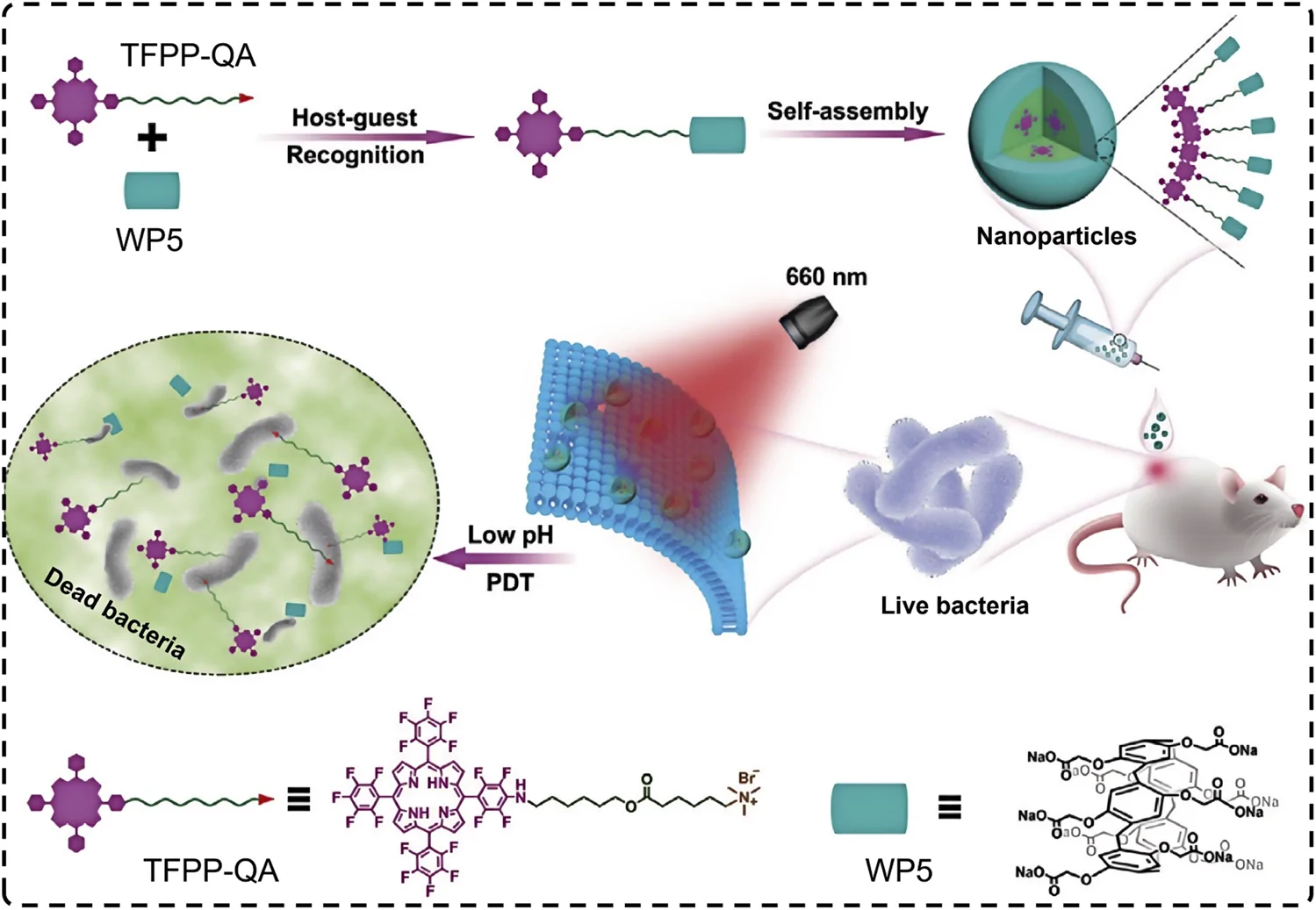
Schematic presentation of an acid-triggered supramolecular photosensitizer and its antibacterial activity under light irradiation. Adapted with permission from Xia et al., 2022.

Infectious diseases caused by pathogenic bacteria have long posed one of the most serious threats to human health and have thus attracted considerable research attention. In 2022, Zhang and co-workers constructed a supramolecular PS platform based on WP5 and a quaternary ammonium-functionalized tetrafluorophenyl porphyrin (TFPP-QA) for antibacterial and biofilm dispersal applications via PDT (Figure 2). Benefiting from the introduction of WP5 and host-guest interactions, this supramolecular PS exhibits favorable biocompatibility and acid-responsive properties. On the one hand, acid-triggered dissociation of the TFPP-QA/WP5 complex facilitates the targeted delivery of the porphyrin PS to bacterial cells, disrupts the charge balance of the bacterial membrane, and enhances membrane permeability. On the other hand, the TFPP-QA/WP5 platform demonstrates excellent reactive oxygen species (ROS) generation capability upon light irradiation, thereby augmenting its photodynamic antibacterial efficacy. Both *in vitro* and *in vivo* studies have confirmed that this supramolecular PS achieves potent antibacterial activity and effective biofilm dispersal under 660 nm irradiation. Collectively, these findings suggest that the rational design and integration of PSs with quaternary ammonium compounds through supramolecular strategies hold great promise for future clinical applications in photodynamic antibacterial therapy (Xia et al., 2022).

**FIGURE 3:**
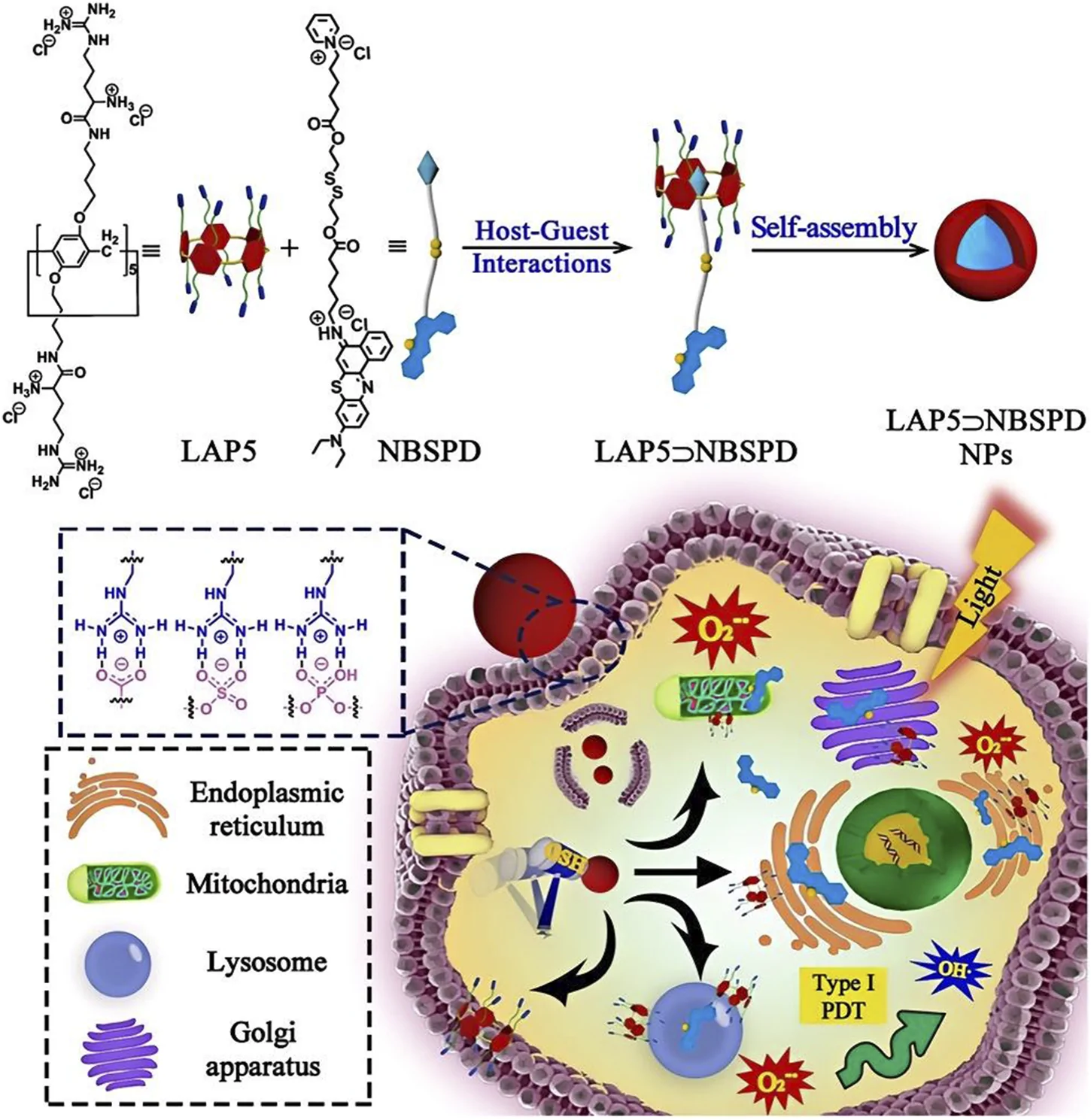
Schematic illustration of the construction of the supramolecular photosensitizer (LAP5⊃NBSPD) and its application for enhanced cancer therapeutic effectiveness. Adapted with permission from Chao et al., 2023.

In addition to carboxylate-modified water-soluble pillar[5]arenes, Prof. Pei’s group designed and synthesized an L-arginine-functionalized water-soluble pillar[5]arene (LAP5) in 2023 (Chao et al., 2023). They constructed a supramolecular PS system through the host–guest interactions between LAP5 and a pyridine derivative of Nile blue analogs (NBSPD). This system integrates LAP5-induced disruption of cancer cell membranes with Type I PDT to achieve synergistically enhanced anticancer efficacy. As illustrated in Figure 3, the amphiphilic supramolecular photosensitizer (LAP5⊃NBSPD) self-assembles into nanoparticles (LAP5⊃NBSPD NPs). The guanidinium groups on the surface of LAP5⊃NBSPD NPs form multiple salt bridges with cancer cell membranes and facilitate cellular internalization via endocytosis. In the presence of elevated GSH levels, both LAP5 and NBS are released from the nanoparticles and subsequently distributed across various organelles, including mitochondria, the endoplasmic reticulum, lysosomes, and the Golgi apparatus. Ultimately, LAP5⊃NBSPD NPs effectively eradicate cancer cells through the synergistic action of reactive oxygen species (O_2_^−^·, H_2_O_2_, and the highly cytotoxic OH) and LAP5-mediated membrane disruption, thereby offering a promising strategy for synergistically enhancing cancer therapeutic outcomes.

**FIGURE 4:**
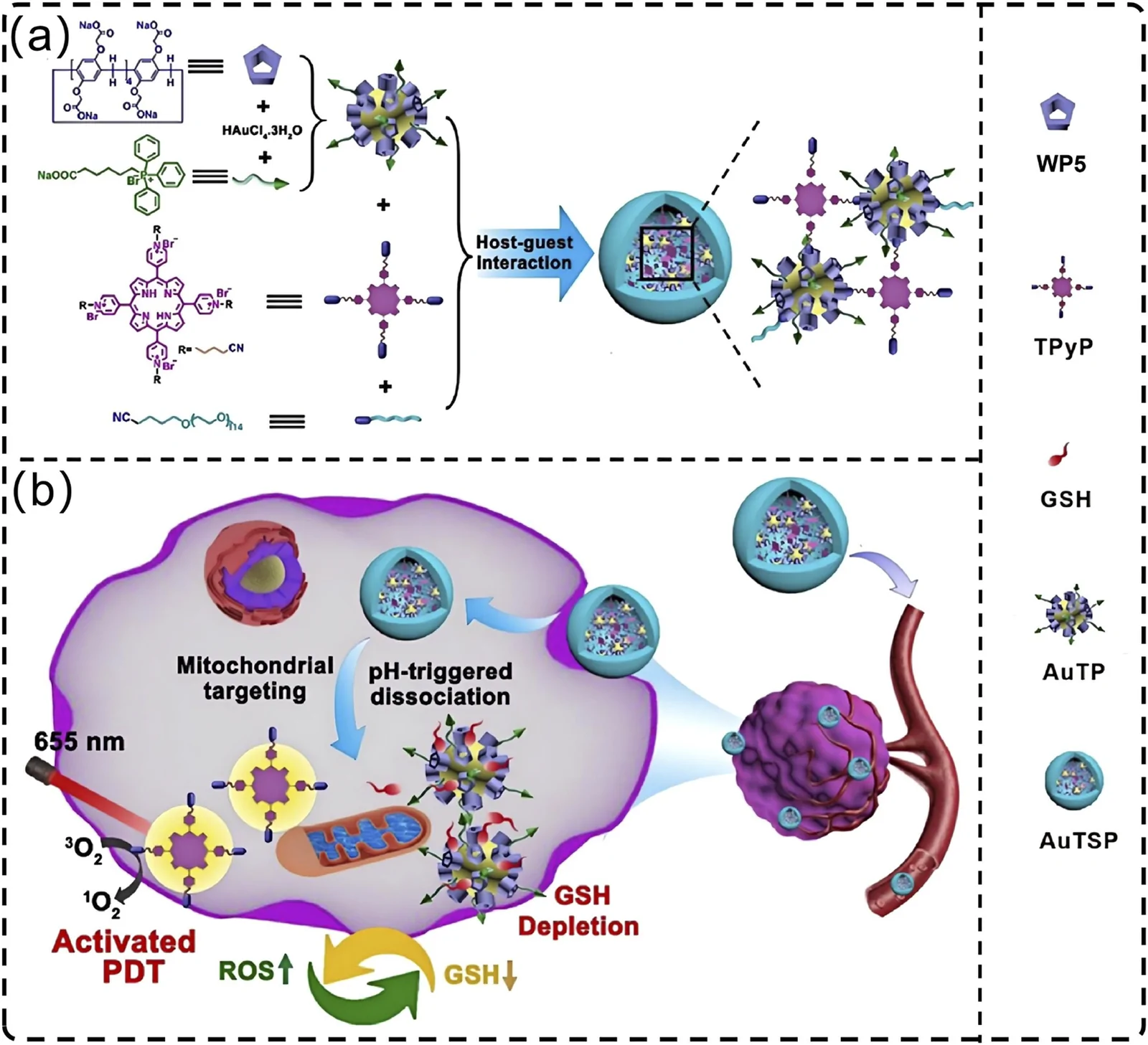
**(a)** Preparation of Mitochondria-Targeting and pH-Switched Supramolecular Photosensitizers (AuTSP) via Host−Guest Interaction between AuTP, TPyP, and PEG in Aqueous Solution. The Photoactivity of TPyP Was Severely Quenched by AuTP. **(b)** Illustration of Self-Amplified and pH-Activated PDT Process. Adapted with permission from Huang et al., 2020.

Carboxylate modification not only endows pillar[n]arenes with water solubility but also serves as an effective stabilizer for gold nanoparticles (AuNPs) (Chen et al., 2019; Yao et al., 2018). On this basis, in 2020, Prof. Zhang’s group proposed a facile and versatile strategy for constructing mitochondria-targeting switchable supramolecular PSs capable of self-amplified, pH-activated PDT. The mitochondria-targeting host motif, denoted as AuTP, was fabricated by co-stabilizing WP5 and (5-carboxypentyl)triphenylphosphonium bromide onto the surface of AuNPs (Huang et al., 2020). Through host–guest interactions, with AuTP as the host motif and tetra(4-*N*-valeronitrile pyridyl)porphyrin (TPyP) together with cyano-terminated poly (ethylene glycol) (PEG) chains as the guest motifs, mitochondria-targeted and pH-switchable supramolecular photosensitizers (AuTSP) were constructed (Figure 4a). The advantages of these hybrid switchable supramolecular photosensitizers for PDT applications are summarized as follows: (I) The dynamic nature of the host–guest interactions, combined with the pH responsiveness of AuTSP, enables precise regulation of the distance between AuTP and TPyP, thereby modulating the FRET effect. This allows the PDT efficacy to be switched off during systemic circulation to minimize off-target toxicity and switched on upon cellular internalization to selectively eradicate cancer cells. (II) The versatile and straightforward functionalization of AuTSP offers a new avenue for the construction of multifunctional PS-based nanoplatforms. (III) The integration of mitochondria-targeting capability with GSH depletion endows AuTSP with the ability to prolong both the lifetime and diffusion radius of generated ROS, particularly singlet oxygen (^1^O_2_), in the vicinity of mitochondria, thereby significantly amplifying the overall PDT efficacy (Figure 4b).

**FIGURE 5:**
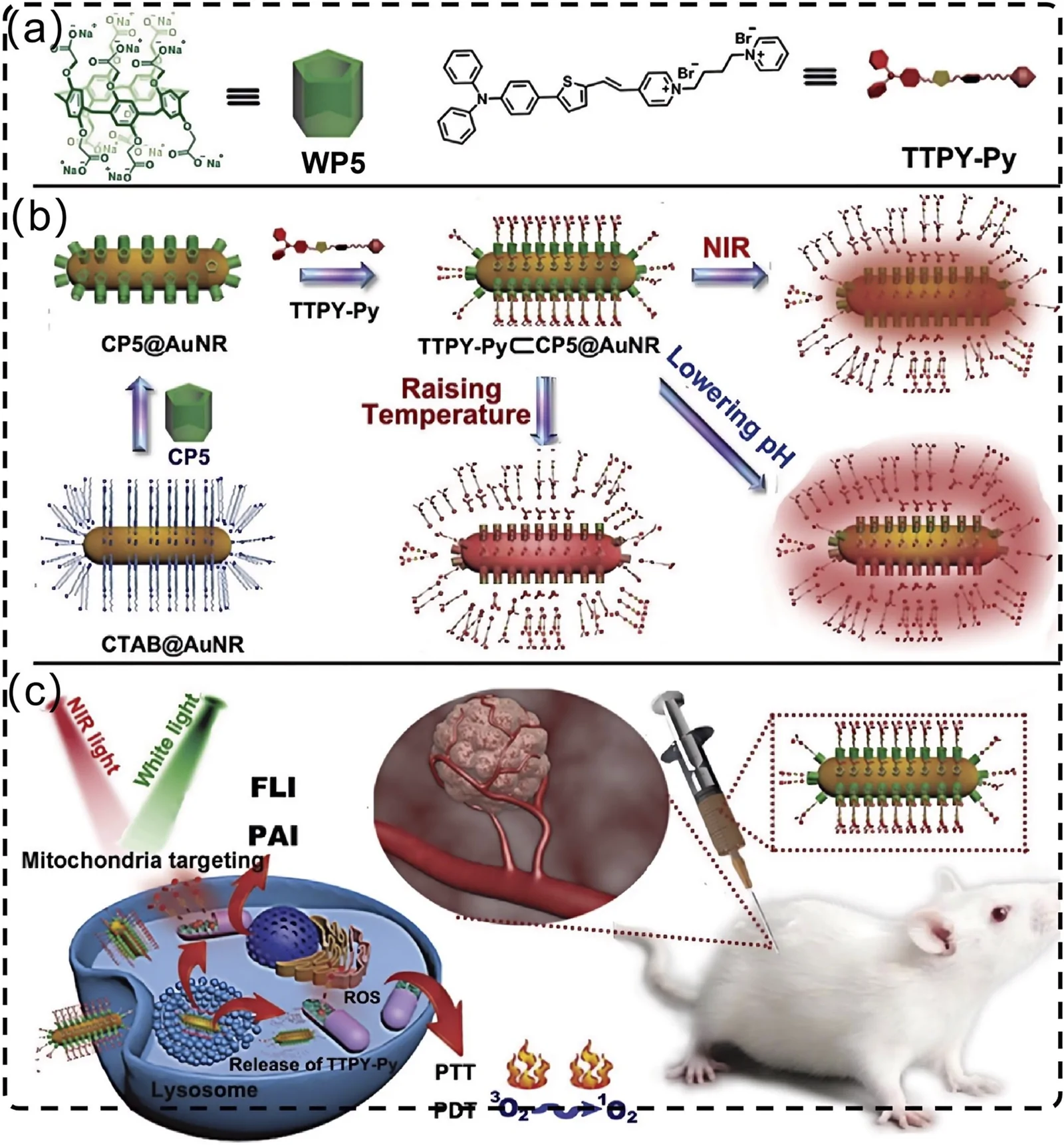
Schematic illustration for the preparation of the nanomaterials with supramolecular assembly of WP5 and TTPY-Py and their application in synergistic photodynamic-photothermal therapy with real-time tumor-imaging. **(a)** Chemical structures of WP5 and TTPY-Py; **(b)** Schematic diagram for preparation of TTPY-Py⊂WP5@AuNR and the multistimuli-responsive properties; **(c)** The theranostic applications of the multifunctional materials for FLI-PAI imaging-guided synergistic PDT-PTT therapies. Adapted with permission from Song et al., 2021.

In addition, the group of Prof. Tang constructed a multifunctional theranostic nanosystem named TTPY-Py⊂WP5@AuNR by employing WP5-stabilized gold nanorods (AuNRs) (Figure 5). Unlike previous studies that relied on cumbersome covalent methods to anchor macrocycles onto AuNR surfaces, the present work adopted a facile ligand-exchange strategy to prepare carboxylatopillar[5]arene (WP5)-modified AuNRs (89. Song et al., 2021). This approach effectively circumvented the intrinsic toxicity of AuNRs, and the resulting AuNRs served as efficient nanocarriers for targeted ligand delivery with the assistance of WP5. Concurrently, an AIE-active photosensitizer (TTPY-Py) bearing pyridinium terminals was synthesized as the guest molecule. WP5@AuNR enabled the delivery of TTPY-Py into cancer cells through host–guest interactions between WP5 and TTPY-Py. Within this hybrid supramolecular nanomaterial, the advantages and functionalities of each component were integrated as follows: i) WP5-stabilized AuNRs exhibited low cytotoxicity and an excellent photothermal effect, making them suitable for photothermal therapy (PTT) and photoacoustic imaging (PAI); ii) the encapsulation and release of TTPY-Py were controllable under triple stimuli, including pH, temperature, and near-infrared (NIR) irradiation; and iii) the released TTPY-Py could selectively target mitochondria by virtue of its positively charged pyridinium moieties while simultaneously generating ROSs for PDT. Consequently, a synergistic PTT–PDT theranostic strategy was achieved, affording enhanced therapeutic efficacy and imaging-guided treatment advantages.

WP5 possesses a compact cavity that optimally accommodates small-to-medium planar cationic guests, effectively suppressing ACQ and enhancing ROS generation via tight encapsulation. The deca-carboxylate modification ensures good water solubility and pH responsiveness, but offers limited functional diversity. WP5-based systems are reliable for solubility improvement and pH-triggered release, yet rely on additional components (e.g., AuNPs, peptides) to achieve targeting or combination therapy, with straightforward preparation but restricted multifunctional integration.

## 3 Supramolecular PDT systems based on monofunctionalized Pillar[5]arene host-guest chemistry

The functionalization of water-soluble pillar[n]arenes has generally been limited to a few types of groups, such as carboxylates, phosphates, quaternary ammonium salts, and glycol chains, while the installation of more complex functionalities onto all phenolic hydroxy groups of the pillar[n]arene scaffold remains challenging. It has been established that selective monodealkylation of the alkoxy groups on pillar[n]arenes affords monophenolic hydroxy-functionalized intermediates, which serve as versatile handles for the facile introduction of various sophisticated moieties, thereby enabling the preparation of a diverse array of monofunctionalized pillar[n]arene derivatives (Sun et al., 2018).

**FIGURE 6:**
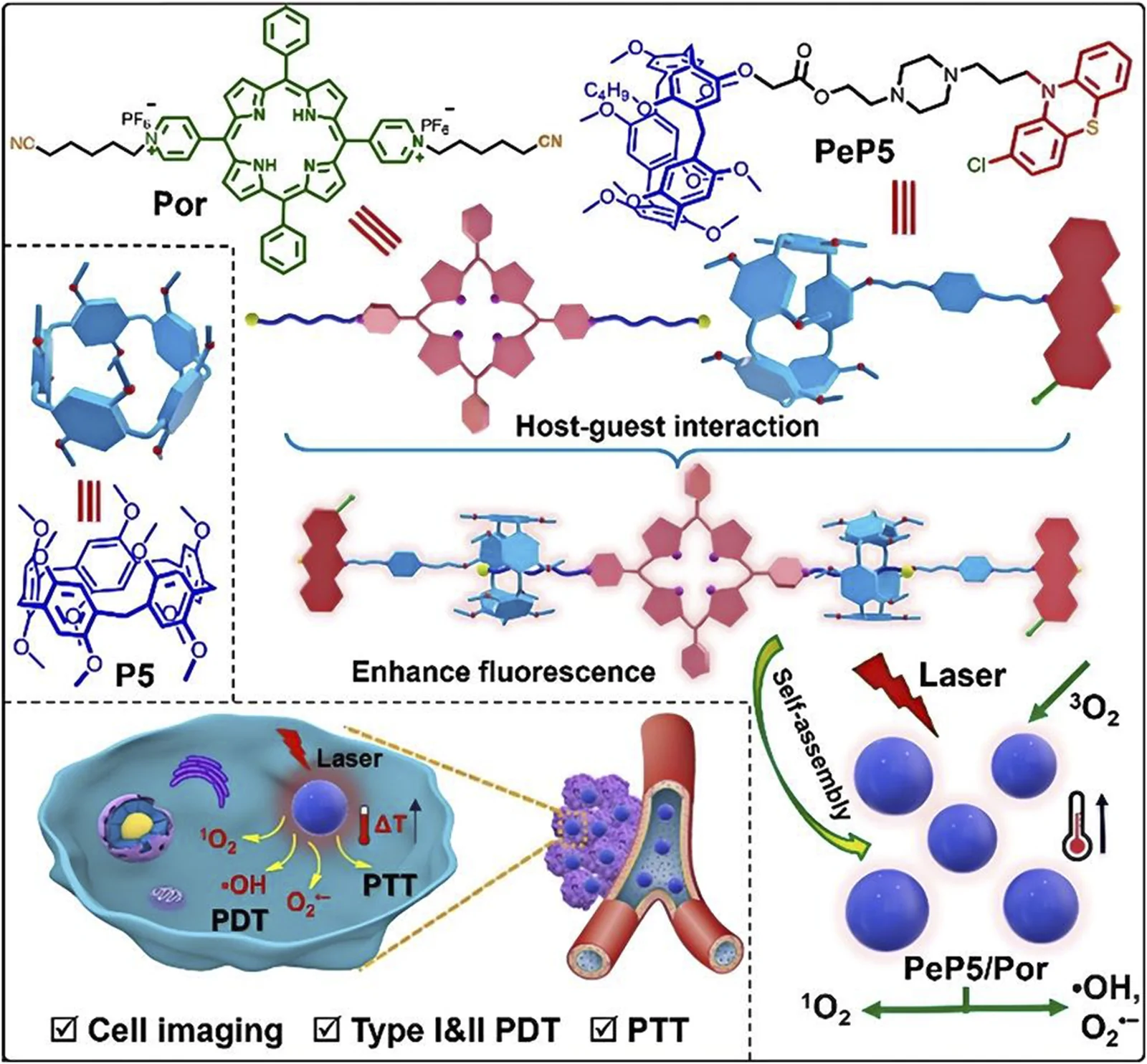
Chemical structures of perphenazine-modified-pillar [5]arene (PeP5), and the porphyrin-guest (Por) and cartoon representations of PeP5⊃Por based nano-assemblies for synergistic photothermal and photodynamic (^1^O_2_, OH, and O_2_^•-^) therapy. Adapted with permission from Ma et al., 2024.

Perphenazine is an antipsychotic agent endowed with antipyretic, antiemetic, and sedative properties, and is clinically indicated for various types of schizophrenia, effectively alleviating hallucinations, delusions, anxiety, tension, and agitation. In 2024, our group synthesized a monofunctionalized pillar[5]arene derivative, designated PeP5, by covalently attaching perphenazine to the pillar[5]arene skeleton (Ma et al., 2024). Concurrently, a two-armed guest porphyrin compound, Por, was prepared by modifying a para-pyridine-functionalized porphyrin with bromohexanenitrile. Utilizing the host–guest recognition between PeP5 and Por, we constructed an efficient photothermal and photodynamic cancer therapeutic platform, denoted as PeP5⊃Por (Figure 6). The resulting supramolecular complex could self-assemble into nanoparticles in aqueous solution via non-covalent interactions. Dynamic light scattering (DLS) measurements revealed a hydrodynamic diameter of approximately 150 nm for the PeP5⊃Por nanoparticles. Scanning electron microscopy (SEM) and transmission electron microscopy (TEM) characterizations further demonstrated that the nanoparticles exhibited well-defined spherical morphology with an average diameter of around 135 nm, a size regime that is advantageous for exploiting the EPR effect for passive tumor targeting. Importantly, the Perphenazine moieties in PeP5 not only facilitated the generation of singlet oxygen (^1^O_2_) and hydroxyl radicals (·OH), but also efficiently produced superoxide anion radicals (O_2_·^-^). Moreover, the PeP5⊃Por assemblies displayed satisfactory photothermal conversion efficiency, enabling combined photothermal/photodynamic therapy. Collectively, this study offers a novel and effective strategy for enhancing the bioactivity of pillar[5]arene-based host–guest complexes through rational molecular modification.

**FIGURE 7:**
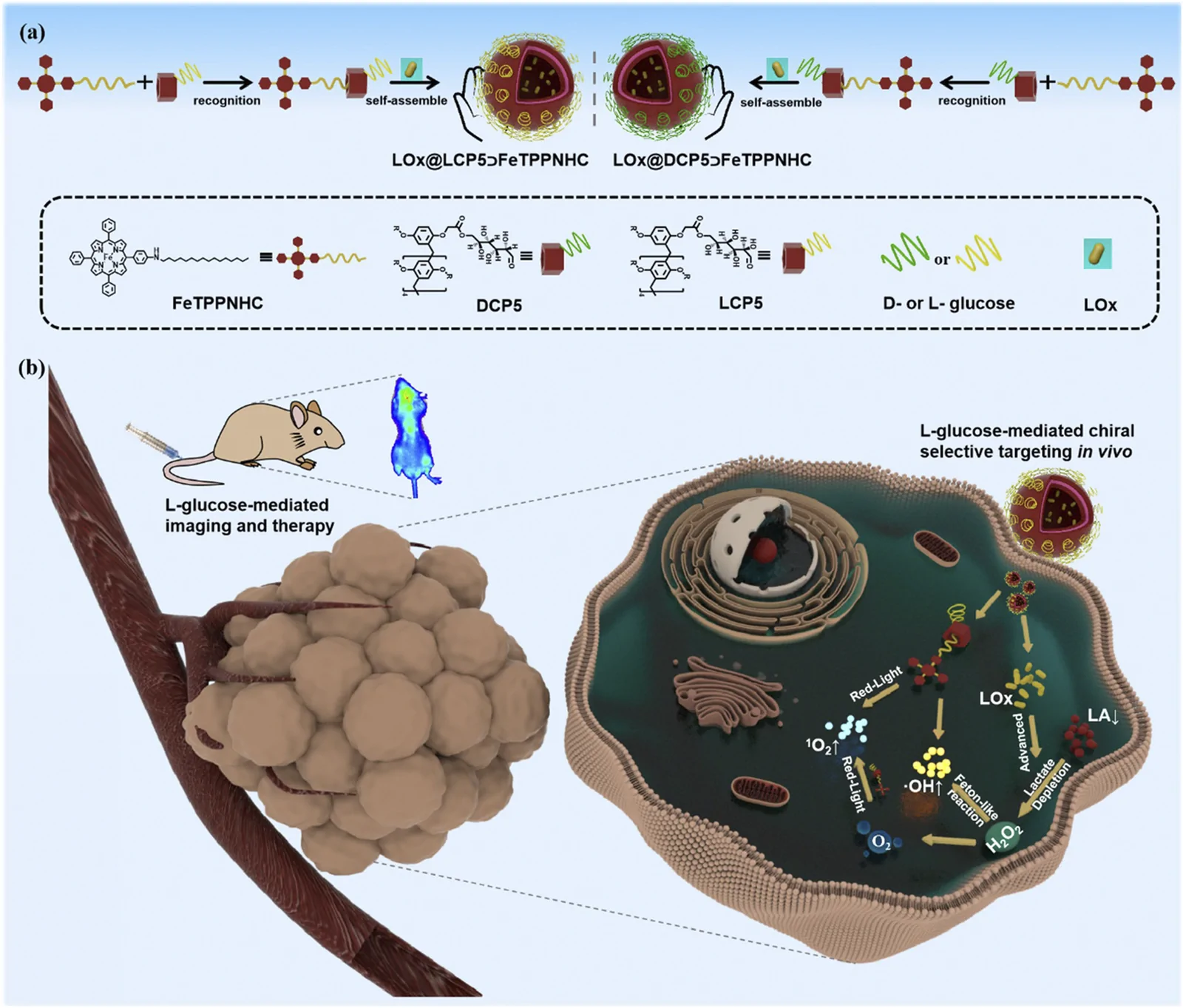
**(a)** An acid-responsive chiral supramolecular vesicle was constructed to transport lactate oxidases through the host–guest interaction of D-/L-glucose-modified pillar [5]arene and Fe-porphyrin derivatives. **(b)** The L-glucose-mediated chiral vesicle demonstrates outstanding chirality recognition and lactate depletion abilities. Both *in vitro* and *in vivo* studies reveal the high tumor specificity and therapeutic efficacy of LOx@LCP5⊃FeTPPNHC, indicating the significant potential of chiral glucose-modified nanomaterials in targeted cancer treatment. Adapted with permission from Zhang et al., 2025b.

The distinct interactions of D/L-glucose with cells and biological systems have attracted widespread research interest. However, the potential application of chiral glucose-modified nanomaterials in cancer diagnosis and therapy remains to be systematically explored. In 2025, Prof. Wang’s group first synthesized D/L-glucose-modified mono-functionalized pillar[5]arene (D-/L-CP5). Subsequently, leveraging the host–guest recognition between D-/L-CP5 (as the host) and iron porphyrin derivatives (FeTPPNHC, as the guest), they successfully constructed an acid-responsive chiral supramolecular vesicle capable of encapsulating and delivering lactate oxidase (LOx) (denoted as LOx@D-/L-CP5⊃FeTPPNHC) (Figure 7). This system was designed to deplete lactate (LA) in the tumor microenvironment, thereby synergistically enhancing chirality-mediated tumor-specific cascade chemodynamic therapy (CDT) and PDT (Zhang Z. et al., 2025). Remarkably, the L-glucose-mediated chiral vesicles exhibited superior chiral recognition and lactate depletion capabilities compared to their D-glucose-mediated counterparts. Once internalized by tumor cells, the L-type supramolecular nanomicelles could directly consume lactate and generate substantial amounts of H_2_O_2_, which were subsequently converted into highly cytotoxic OH and ^1^O_2_. Both *in vitro* and *in vivo* experiments confirmed the excellent tumor specificity and therapeutic efficacy of LOx@L-CP5⊃FeTPPNHC. These findings indicate that chiral glucose-modified nanomaterials hold great promise for targeted cancer therapy, paving the way for the development of innovative anticancer strategies based on their unique interactions with biological systems.

**FIGURE 8:**
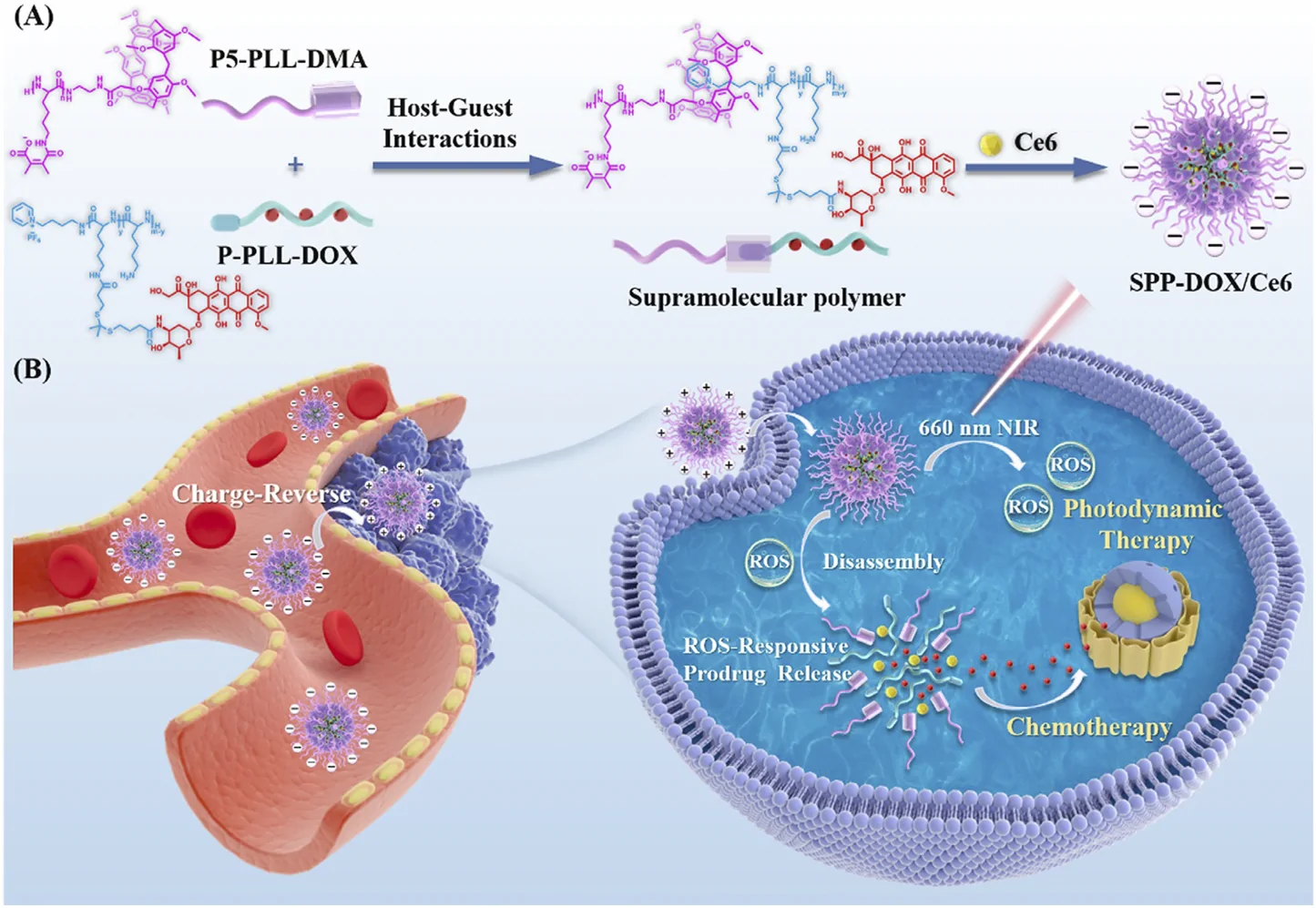
**(A)** Schematic illustration of the fabrication of supramolecular polymer and Ce6-loaded nanoparticles SPP-DOX/Ce6; **(B)** the mechanism of charge-reverse and PDT-CT combination therapy of SPP-DOX/Ce6. Adapted with permission from Ding et al., 2022a.

We further developed a charge-reversal amphiphilic pillar[5]arene functionalized with a single polypeptide chain (P5-PLL-DMA), along with a reactive oxygen species (ROS)-responsive polypeptide prodrug (P-PLL-DOX), in which doxorubicin (DOX) was covalently conjugated to poly (L-lysine) (PLL) via an ROS-cleavable thioketal (TK) linker. These two components could undergo supramolecular assembly through pillar[5]arene-based host–guest recognition, and further encapsulate the photosensitizer chlorin e6 (Ce6), affording a supramolecular polypeptide prodrug nanosystem (SPP-DOX/Ce6) (Figure 8). In this system, DOX was loaded via chemical conjugation, and the negatively charged surface of SPP-DOX/Ce6 not only effectively prevented premature drug leakage but also minimized nonspecific interactions with serum proteins, thereby substantially enhancing its colloidal stability under physiological conditions (pH 7.4). Upon exposure to the acidic tumor microenvironment (pH 6.5), the surface charge of SPP-DOX/Ce6 underwent a reversal from negative to positive, which facilitated cellular internalization and enabled efficient intracellular delivery of DOX. Furthermore, upon irradiation with 660 nm NIR light, the encapsulated Ce6 generated ROS that rapidly cleaved the TK linker, releasing the activated DOX and achieving tumor-specific drug release (Ding et al., 2022a).

**FIGURE 9:**
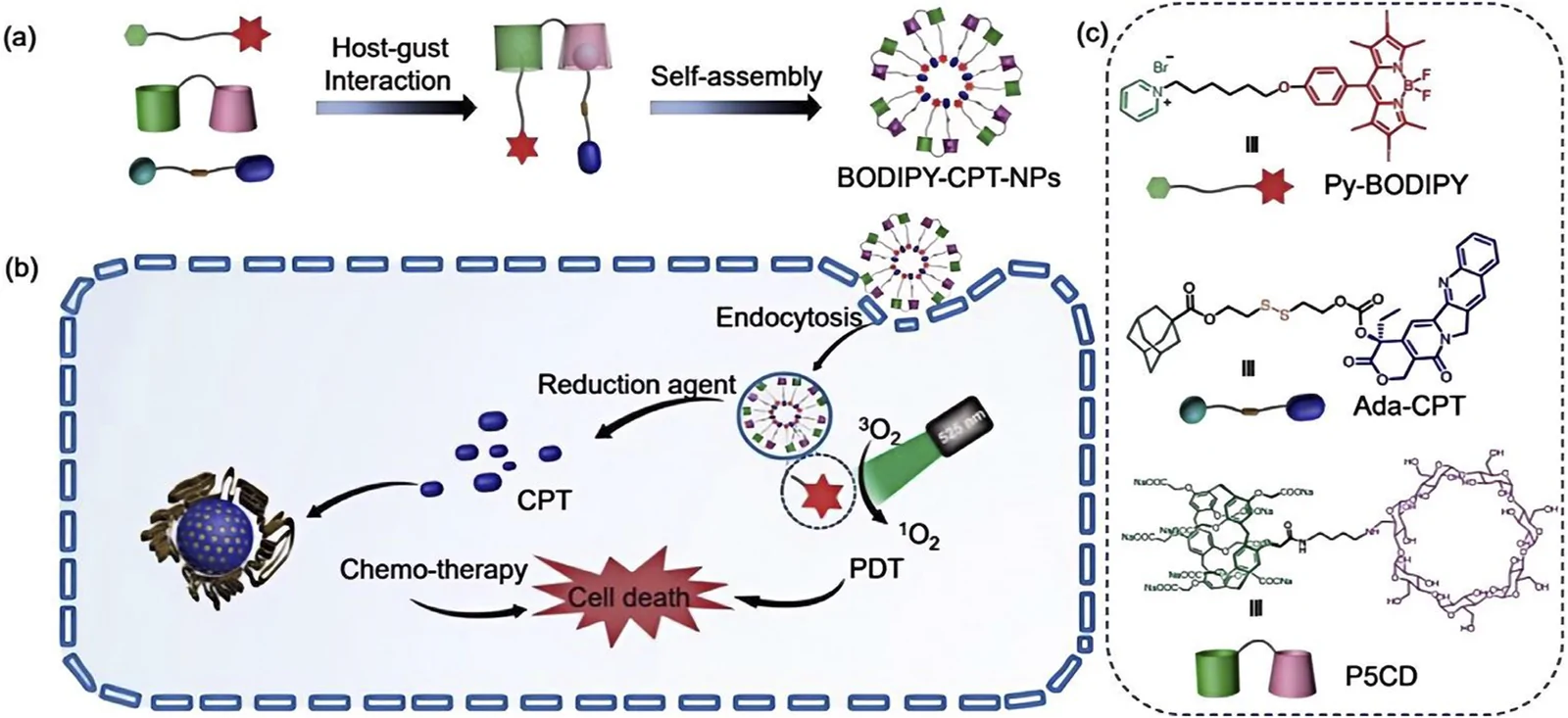
**(a)** Preparation pathway of BODIPY-CPT-NPs via host-guest interactions and self-assembly; and **(b)** a schematic illustration of the mechanism of chemophotodynamic combination therapy by BODIPY-CPT-NPs. **(c)** Chemical structures of Py-BODIPY, Ada-CPT, and P5CD. Adapted with permission from Yin et al., 2023.

Of particular interest, the group of Prof. Wang designed a dual-macrocyclic host molecule (P5CD) by functionalizing pillar[5]arene with a cyclodextrin (CD) moiety, and further exploited its host–guest recognition properties to construct a novel type of supramolecular nanoparticles for combined chemo-photodynamic therapy (Yin et al., 2023; Figure 9). These nanoparticles are composed of three functional components: (1) the dual-macrocyclic host P5CD, which features both WP5 and β-CD binding sites; (2) the photosensitizer Py-BODIPY, decorated with a pyridinium salt unit; and (3) the prodrug Ada-CPT, in which adamantane is covalently conjugated to the chemotherapeutic agent camptothecin (CPT) via a redox-responsive disulfide linker.

The construction strategy relies on orthogonal host–guest recognition—a design principle in which two chemically distinct recognition motifs operate independently without mutual interference. The WP5 site selectively binds the cationic pyridinium group of Py-BODIPY, while the β-CD site binds the adamantane moiety of Ada-CPT. These two binding interactions are chemically orthogonal: the adamantane group is too bulky for the WP5 cavity, and the β-CD cavity does not effectively accommodate the pyridinium cation. This intrinsic selectivity prevents competitive binding between the two functional modules, ensuring that each P5CD molecule simultaneously hosts one Py-BODIPY and one Ada-CPT.

Through orthogonal host–guest recognition, the three components assemble into a ternary complex with precisely defined 1:1:1 stoichiometry. The ternary complexes subsequently self-assembles into supramolecular nanoparticles (designated as BODIPY-CPT-NPs). Owing to the well-defined stoichiometry of the orthogonal recognition pairs, the loading ratio of the prodrug to the photosensitizer within the nanoparticles can be precisely tuned as required by adjusting the feed ratio of the three components during formulation—a critical advantage over conventional co-encapsulation strategies that lack such stoichiometric control. As illustrated in Figure 9b, upon cellular internalization of BODIPY-CPT-NPs, the disulfide bond in the Ada-CPT moiety is cleaved by the intracellular reductive environment, leading to the release of active CPT for chemotherapy. Concurrently, upon light irradiation, Py-BODIPY efficiently generates ^1^O_2_ for photodynamic therapy. Cytotoxicity assays demonstrated that BODIPY-CPT-NPs exhibit a remarkable synergistic effect in chemo-photodynamic combination therapy.

MP5 retains the same cavity size as WP5 while incorporating a single bioactive functional handle (e.g., perphenazine, glucose, polypeptides) at one rim. This asymmetric design preserves high-affinity guest recognition and ROS production, while introducing supplementary therapeutic actions such as membrane disruption, chiral discrimination, or charge reversal. The trade-off is more demanding synthesis, yet MP5 offers a versatile strategy for converting simple hosts into multifunctional theranostic agents beyond pure PDT.

## 4 Supramolecular PDT systems based on Pillar[6]arene host-guest chemistry

**FIGURE 10:**
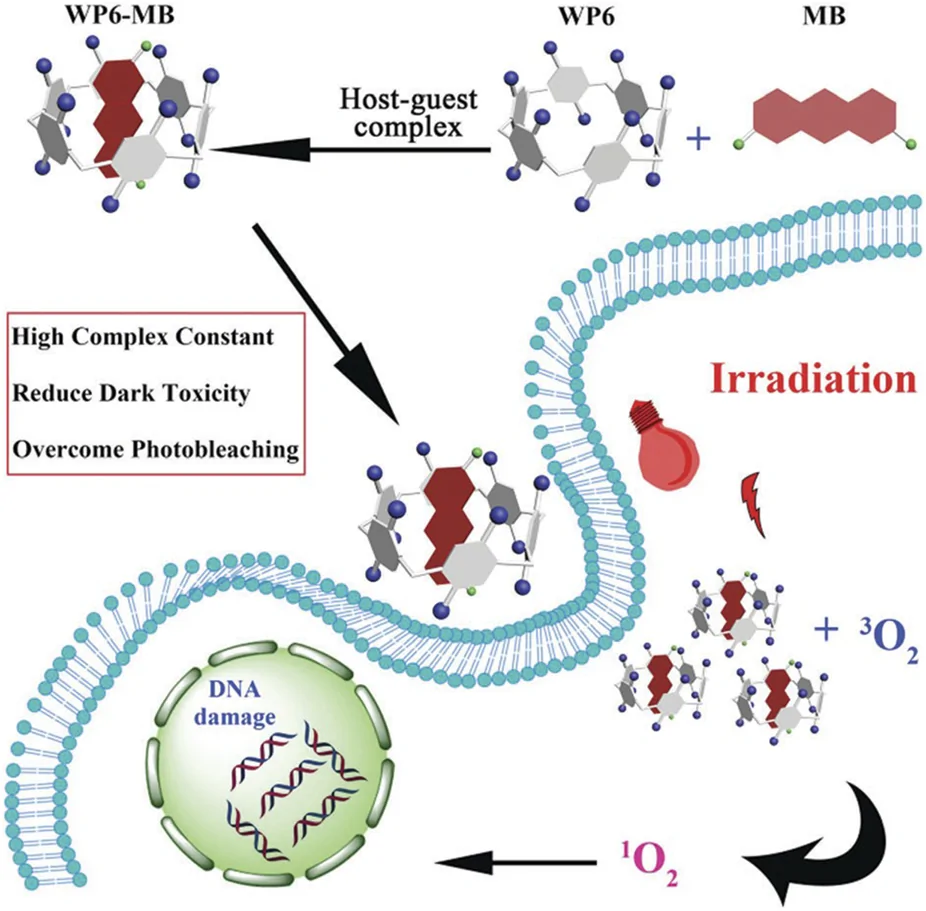
Schematic of the construction of the supramolecular photosensitizer system based on the host–guest complexation between WP6 and MB for PDT. Adapted with permission from Yang et al., 2018.

Pillar[6]arenes possess a larger cavity compared to pillar[5]arenes (Cao et al., 2022), enabling the encapsulation of bulkier guest molecules and thereby facilitating enhanced generation of ROSs. In this context, the group of Prof. Pei designed and synthesized a carboxylate-modified water-soluble pillar[6]arene (WP6), and further exploited its host–guest interactions with the photosensitizer methylene blue (MB) to construct a supramolecular photosensitizer system (WP6–MB) for sustained PDT (Figure 10) (Yang et al., 2018). MB can directly complex with WP6 without the need for any chemical modification, forming a 1 : 1 host–guest complex with an association constant of (1.15 ± 0.30) × 10^7^ M^-1^, as confirmed by ^1^H NMR, UV–vis absorption spectroscopy, and fluorescence emission spectroscopy. Compared with free MB, the WP6–MB complex retains its photodynamic activity while exhibiting two notable advantages. On one hand, complexation with WP6 effectively reduces the dark toxicity of MB, which has long been recognized as one of the major obstacles to the clinical application of PDT. On the other hand, this system significantly suppresses the photo bleaching of MB and prolongs the duration of ROS generation under continuous light irradiation, thereby enhancing the overall PDT efficacy. These advantages were further corroborated by *in vitro* ROS generation assays and cytotoxicity evaluations against a panel of cell lines, including MCF-7, HeLa, HepG2, HL7702, and 293T cells.

**FIGURE 11:**
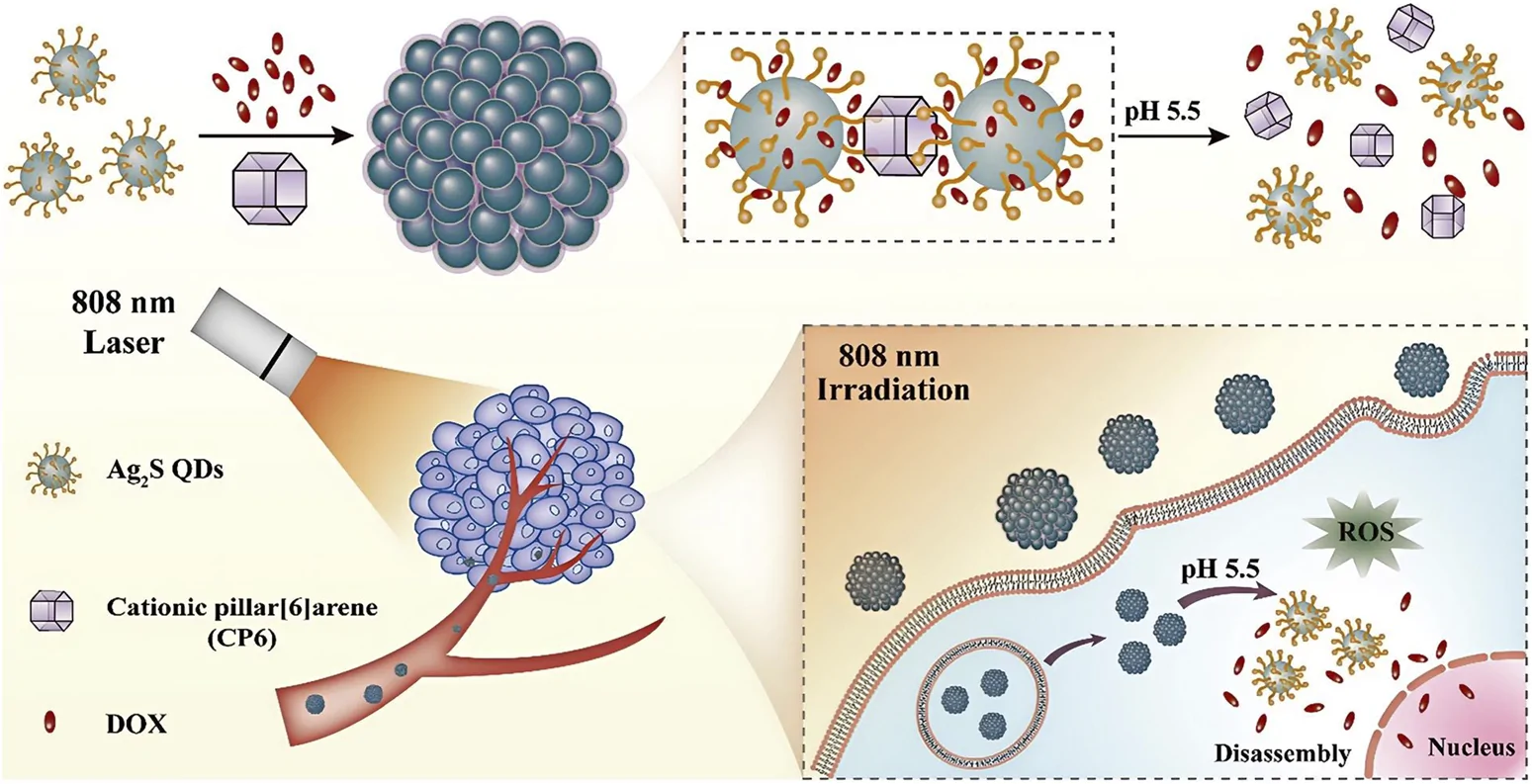
A representation of the self-assembled co-delivery system (Ag_2_S-DOX-CP6) based on host-guest interaction for chemo-photodynamic synergistic therapy of cancer. Adapted with permission from Wu et al., 2023.

The research group of Professor Li. developed a self-assembled co-delivery system (Ag_2_S-DOX-CP6) based on quaternary ammonium-modified water-soluble pillar[6]arene (CP6), with the aim of enhancing PDT efficiency and achieving stimuli-responsive drug release for combined chemo-photodynamic cancer therapy (Wu et al., 2023). By leveraging host–guest interactions, the group successfully engineered the self-assembly of Ag_2_S quantum dot (QD) PSs with CP6 into ordered nanostructures (Figure 11). In this process, the high surface-area-to-volume ratio of Ag_2_S QDs was exploited to generate ample hydrophobic spaces for encapsulating DOX molecules, achieving a drug-loading efficiency of 10.2%. In phosphate-buffered saline at pH 5.5, the cumulative DOX release reached 43.1% and 52.1% after 24 h and 48 h, respectively, indicating the system’s capability for intelligent drug release within the acidic microenvironment of tumor cells. Furthermore, Ag_2_S QDs exhibited high catalytic activity under NIR irradiation, and their outstanding PDT performance was corroborated by both extracellular and intracellular ROSs generation assays. Ag_2_S QDs served dual functions as PSs to generate ROS under 808 nm NIR light and as nanocarriers to improve the delivery efficiency of hydrophobic anticancer drugs to tumors. Uptake evaluations based on confocal laser scanning microscopy (CLSM) images and flow cytometry revealed greater accumulation of Ag_2_S-DOX-CP6 nanoparticles in MCF-7 cells, where they were internalized and subsequently released DOX into the nuclei, thereby achieving effective chemotherapy (CHT). Moreover, when combined with NIR light irradiation, the Ag_2_S-DOX-CP6 treatment reduced MCF-7 cell viability to 8.8%, compared with 49.91% for the free DOX group at an equivalent DOX concentration of 2 mg mL^-1^. The *in vivo* antitumor efficacy and biocompatibility of Ag_2_S-DOX-CP6 were confirmed in animal models. Hematoxylin and eosin (H&E) staining and hematological analyses of major organs from mice indicated low systemic toxicity and satisfactory biocompatibility of the co-delivery system. Collectively, these findings suggest that the Ag_2_S-DOX-CP6 co-delivery system holds great promise as a drug delivery platform for chemo-photodynamic cancer therapy, capable of co-delivering PSs and therapeutic agents to tumor sites while exerting a synergistic chemo-photodynamic therapeutic effect.

WP6 features a larger cavity than WP5, enabling encapsulation of bulkier planar guests (e.g., methylene blue) without pre-modification. The spacious interior better stabilizes PS excited states, suppresses photobleaching, and sustains ROS generation over prolonged periods, while reducing dark toxicity. However, increased cavity size brings greater conformational flexibility and slightly diminished binding selectivity. WP6 is thus ideal for applications requiring durable photodynamic activity, albeit with modest synthetic challenges.

## 5 Supramolecular PDT systems based on [2]Biphenyl-extended-pillar[6]arene host-guest chemistry

**FIGURE 12:**
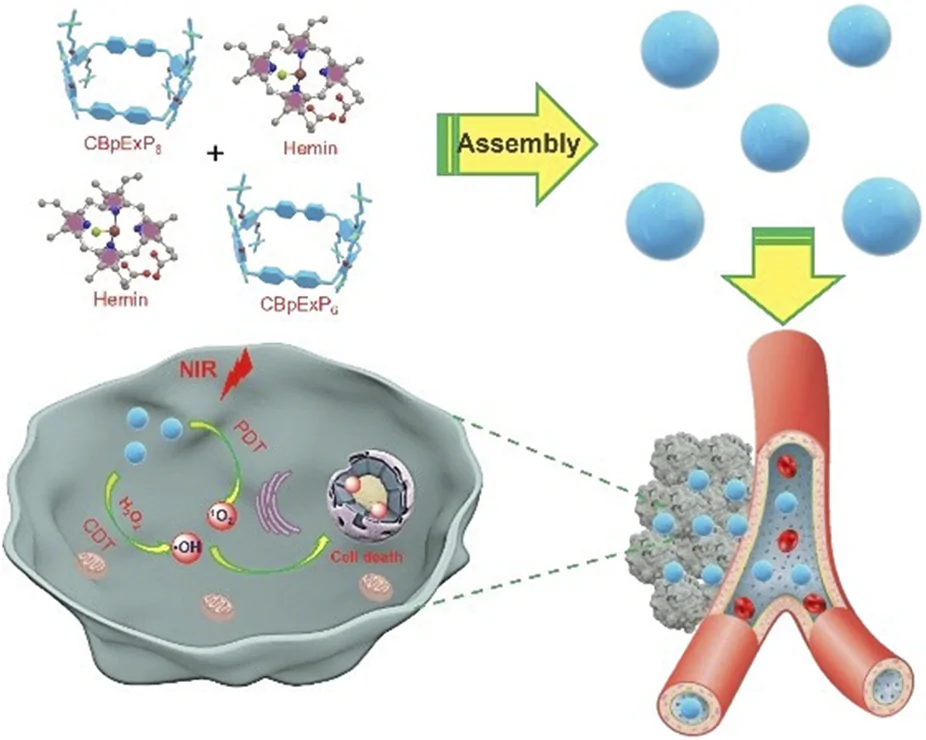
Schematic illustration of hemin self-assembly into nanoparticles in the presence of CBpExP6 and application in cancer therapy. Adapted with permission from Cai et al., 2025c.

Compared with traditional pillar[6]arene, [2]biphenyl-extended pillar[6]arene (ExP6) offers a larger cavity, less crowded substituents on the macrocyclic backbone, and a more efficient synthetic route (Gao et al., 2016). Hemin, a porphyrin derivative bearing two carboxyl groups, exhibits exceptional intestinal absorbability and is recognized as the most bioavailable iron source for human supplementation. Nevertheless, its practical application is hampered by photodegradation upon exposure to light. To address this challenge, we designed and synthesized a novel cationic [2]biphenyl-extended pillar[6]arene derivative (CBpExP6) featuring eight quaternary ammonium functionalities. Isothermal titration calorimetry demonstrated that CBpExP6 forms a stable 1:1 inclusion complex with hemin in aqueous media, substantially enhancing the photostability of the bound hemin (Figure 12). Notably, the resulting complex further self-assembles into well-defined nanoparticles with an average hydrodynamic diameter of approximately 200 nm, which facilitates efficient cellular internalization by tumor cells. Functionally, the ferrous center within the CBpExP6⊃hemin complex catalyzes the conversion of endogenously overexpressed H_2_O_2_ in the tumor microenvironment into highly cytotoxic hydroxyl radicals (·OH). Concurrently, upon light irradiation, the porphyrin core of the complex generates ^1^O_2_ via photosensitization. The synergistic interplay between these two reactive oxygen species markedly amplifies the therapeutic efficacy against malignant cells (Cai et al., 2025c). Collectively, this study offers a promising supramolecular platform for improving PS stability and enhancing photodynamic therapeutic outcomes in anticancer applications.

**FIGURE 13:**
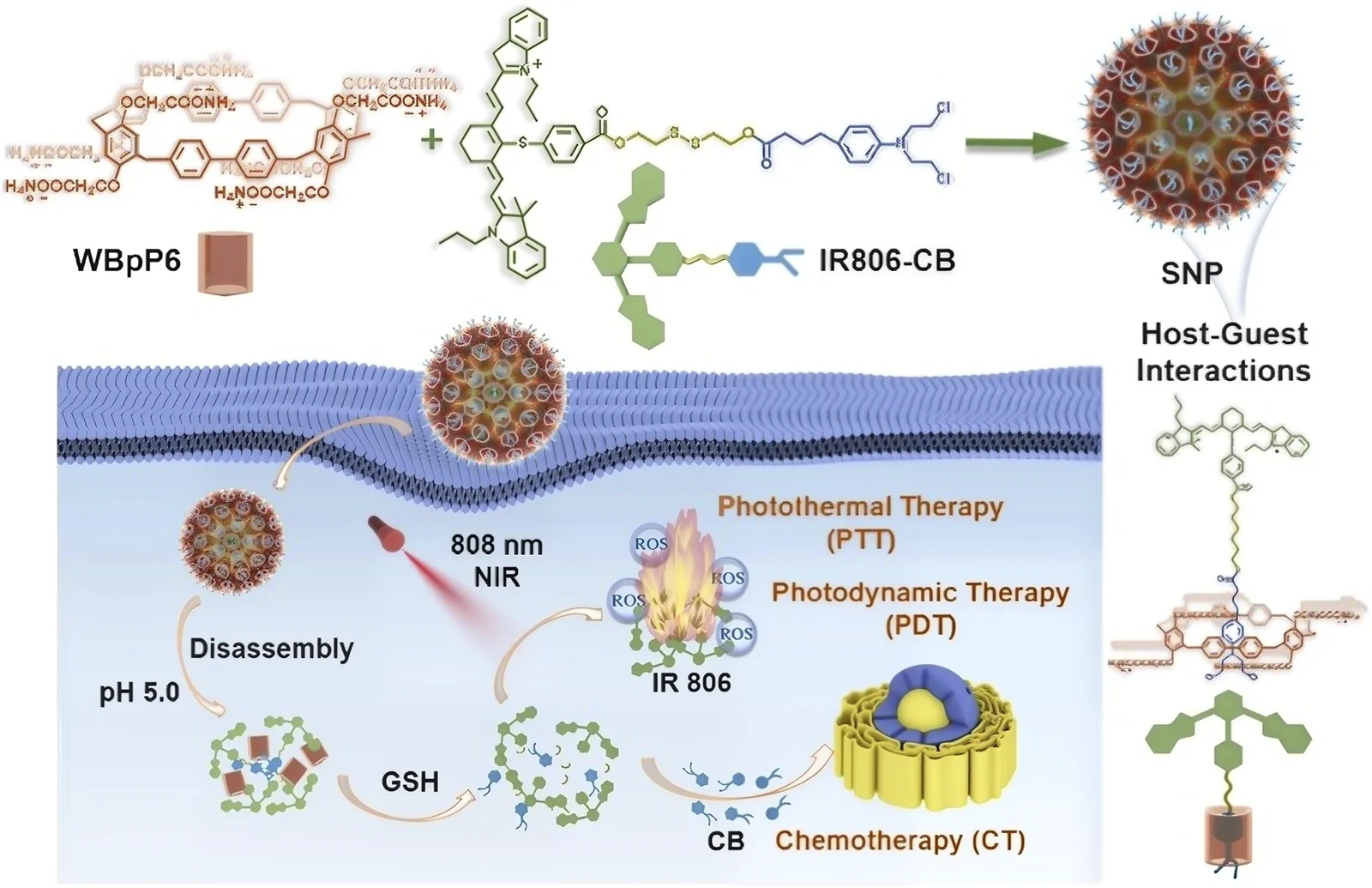
Schematic illustration of the fabrication of supramolecular nanoprodrug SNP and the mechanism of PDT-PTT-CT combination therapy. Adapted with permission from Ding et al., 2022b.

In addition to the ammonium salt-functionalized [2]biphenyl-extended-pillar[6]arene, our group has also conducted a series of studies on the carboxylate-functionalized analogue (WBpP6) for PDT applications. In 2022, we successfully synthesized WBpP6, which exhibits higher synthetic yield and a larger cavity size on the nanometer scale, thereby offering greater potential for encapsulating a wider variety of anticancer drugs (Ding et al., 2022b). Subsequently, a drug–drug conjugate guest (IR806-CB) was obtained by coupling the chemotherapeutic agent chlorambucil (CB) with the NIR heptamethine cyanine dye IR806 via a disulfide linkage, which is cleavable by the elevated glutathione levels within tumor cells (Figure 13). Taking advantage of the host–guest molecular recognition between WBpP6 and CB, we fabricated a supramolecular nanoprodrug (SNP) using WBpP6 and IR806-CB, achieving high drug-loading contents of 25.1% for CB and 76.3% for IR806. Under NIR irradiation (808 nm, 1.0 W/cm^2^, 5 min), the resulting SNP exerted a synergistic triply combined therapeutic effect—photodynamic therapy, photothermal therapy, and chemotherapy (i.e., PDT–PTT–CT)—against HeLa tumor cells.

**FIGURE 14:**
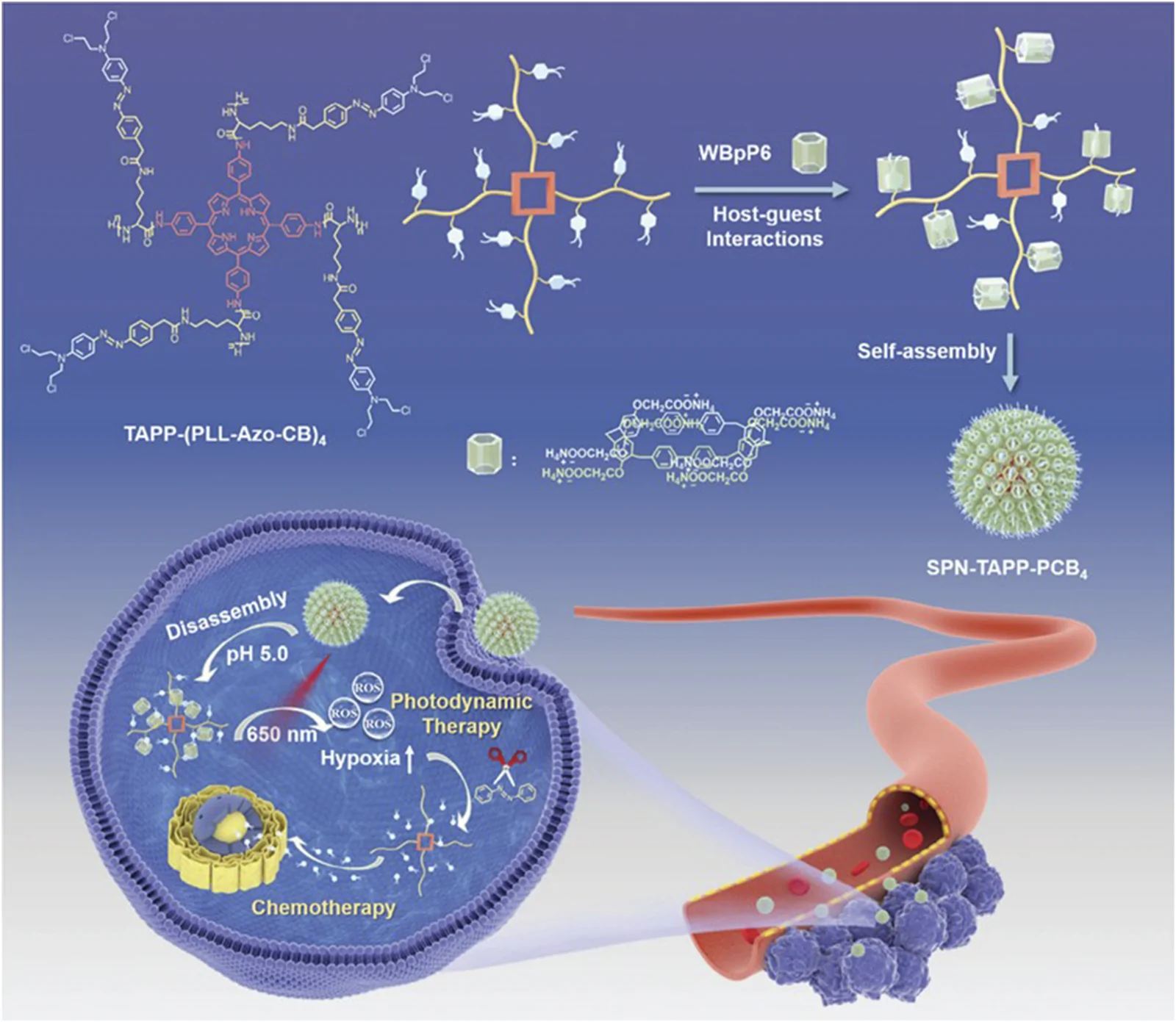
Schematic illustration for the fabrication of hypoxia-responsive tetrameric supramolecular polypeptide nanoprodrug SPN-TAPP-PCB4 and the mechanism of PDT-CT. Adapted with permission from Ding et al., 2024.

In addition, we constructed a hypoxia-responsive tetrameric supramolecular polypeptide nanoprodrug (SPN-TAPP-PCB4) via the self-assembly of WBpP6 with a tetraphenylporphyrin-centered poly (L-lysine-azobenzene-chlorambucil) conjugate (TAPP-(PLL-Azo-CB)_4_), driven by the synergistic effects of hydrophobic interactions, π–π stacking, and host–guest recognition (Figure 14) (Ding et al., 2024). Upon laser irradiation, the central TAPP moiety efficiently converts oxygen into ^1^O_2_, thereby exerting potent cytotoxicity against tumor cells. Moreover, under acidic and PDT-aggravated hypoxic tumor microenvironments, SPN-TAPP-PCB4 undergoes rapid disassembly, followed by the hypoxia-triggered cleavage of azobenzene linkages, leading to the controlled release of activated chlorambucil (CB). Both *in vitro* and *in vivo* biological evaluations demonstrated that this system exhibits a significant synergistic anticancer effect in the combined PDT and chemotherapy (CT) regimen, with negligible systemic toxicity. Collectively, this supramolecular polypeptide nanoprodrug platform offers a promising strategy for designing hypoxia-responsive nanocarriers toward synergistic PDT-CT combination therapy. In a related approach, Prof. Chen. and coworkers exploited the host-guest chemistry of WBpP6 with dendritic drug-drug conjugate guests to construct hypoxia-responsive supramolecular nanoparticles for synergistic PDT-PTT-CT combination therapy (Ling et al., 2025).

ExP6 offers an even larger nanoscale cavity with reduced steric hindrance and improved synthetic accessibility. This allows accommodation of bulky guests (e.g., hemin, dendritic conjugates) and enables synergistic PDT/CDT via Fenton-like reactions, along with high drug loading and multimodal combination therapy (PDT-PTT-CT). Disadvantages include greater molecular weight and complex stoichiometry. ExP6 stands out as a robust scaffold for multifunctional combination nanoplatforms where large-guest encapsulation and therapeutic synergy are prioritized.

## 6 Conclusions

This review systematically summarizes the applications of pillar[n]arene host-guest chemistry in photodynamic therapy (PDT), covering the evolution of host skeletons, molecular recognition mechanisms, multifunctional integration strategies, and biomedical implementations. Benefiting from rigid, symmetric cylindrical cavities, facile structural derivatization, and versatile host–guest recognition performance, pillar[n]arenes have evolved from structurally novel macrocycles into core building blocks for supramolecular photosensitizers with tunable functionality and stimulus-controllable responsiveness.

Water-soluble pillar[5]arenes were first exploited to encapsulate cationic or neutral photosensitizers. The cavity shielding effect efficiently suppresses π–π stacking aggregation, remarkably elevating fluorescence quantum yields and ROSs generation efficiency. Further conjugation with functional guests such as gold nanoparticles and peptide prodrugs yields multifunctional nanoplatforms integrating PDT, PTT, CT, and bioimaging. The advent of monofunctionalized pillar[5]arenes enables a paradigm shift from homogeneous modification to site-specific functionalization, allowing precise installation of intrinsically bioactive moieties including perphenazine, glucose, and peptide chains. The resulting assemblies acquire smart responsive behaviors such as membrane disruption, chiral discrimination, and charge reversal. Owing to enlarged internal cavities, pillar[6]arenes exhibit superior encapsulation capacity for larger planar guests. Their spacious hydrophobic cavities stabilize the excited states of photosensitizers and suppress nonradiative relaxation pathways, thereby boosting photodynamic conversion efficiency. Biphenyl-extended pillar[6]arenes further break the limitation of cavity dimensions; their nanoscale cavities can accommodate biomacromolecules such as heme, accompanied by improved synthetic accessibility and structural tunability. These macrocycles simultaneously enhance photosensitizer stability and realize synergistic PDT/CDT mediated by Fenton-like reactions.

In terms of therapeutic strategy evolution, pillar[n]arene-based supramolecular PDT systems have advanced from simple host encapsulation for ameliorating photosensitizer performance to modular functional integration driven by host–guest recognition. Orthogonal recognition and multicomponent coassembly strategies permit programmable combination of diverse functional motifs to achieve synergistic therapeutic effects among PDT, CT, PTT, and CDT. Stimuli-responsive designs have also expanded from single pH responsiveness to cascade responses toward multiple triggers, including glutathione, ROS, enzymes, hypoxia, and NIR light, which drastically improves spatiotemporal precision of drug release. Practically, such systems have validated potent efficacy in solid tumor ablation and elimination of drug-resistant bacterial infections, alongside favorable biocompatibility and low systemic toxicity. The integration of pillar[n]arene host–guest chemistry and PDT not only offers systematic supramolecular strategies to address inherent drawbacks of conventional PSs, but also opens a vibrant avenue for the design of smart responsive nanomedicines under the framework of precision medicine.

Looking forward, we propose three strategic directions that warrant particular attention. First, targeted functionalization of pillararene-based supramolecular systems remains a critical challenge. While passive targeting via the enhanced permeability and retention (EPR) effect has been widely exploited, active targeting strategies—such as the conjugation of tumor-recognizing ligands (e.g., folic acid, peptides, aptamers, or glycan moieties) to pillararene hosts or guest molecules—hold promise for achieving precise tumor-specific delivery and minimizing off-target toxicity. Moreover, the development of tissue- or cell-type-specific pillararene derivatives through rational cavity modification or peripheral functionalization could further expand the therapeutic window of PDT. Second, multi-modal synergy beyond the currently established combination therapies (e.g., chemo-PDT, photothermal-PDT) represents an underexplored frontier. The modular and orthogonal nature of pillararene-based assembly renders these systems ideally suited for integrating three or more therapeutic and imaging functions—for example, concomitant chemotherapy, PDT, photothermal therapy, immunotherapy, and real-time fluorescence/magnetic resonance imaging—within a single supramolecular nanoplatform. Achieving such “all-in-one” theranostic agents requires not only the careful selection of orthogonal host–guest pairs with mutually non-interfering binding affinities, but also the precise stoichiometric control over multiple functional components, as discussed above. Moreover, the incorporation of stimuli-responsive linkers that respond to distinct pathological cues (pH, GSH, ROS, enzymes, hypoxia) could enable sequential or cascaded drug release, thereby realizing temporally programmed combination therapy that adapts to the dynamic evolution of tumors. Third, the development of degradable macrocyclic skeletons is of paramount importance for clinical translation. Although pillar[n]arenes exhibit excellent host–guest properties and biocompatibility, their non-degradable aromatic framework raises concerns regarding long-term accumulation and potential chronic toxicity *in vivo*. Future efforts should therefore be directed toward the design of biodegradable pillararene analogues—for instance, by introducing cleavable linkages (e.g., ester, disulfide, or acetal groups) into the macrocyclic backbone, or by constructing supramolecular assemblies that disassemble into renally clearable small molecules upon completing their therapeutic mission. Such degradable architectures would not only mitigate safety concerns but also facilitate the regulatory approval process by addressing the “clean clearance” requirement for nanomedicines.

In closing, we envision that the convergence of pillararene chemistry with precision nanomedicine will continue to accelerate, driven by the collective efforts of supramolecular chemists, materials scientists, pharmacologists, and clinicians. With sustained advances in targeted functionalization, multi-modal therapeutic synergy, and biodegradable macrocyclic design, pillararene-based supramolecular photosensitizers are well positioned to evolve from promising research tools into clinically viable therapeutic modalities, ultimately delivering the long-anticipated benefits of precision PDT to patients.
